# Prediction of Chinese suitable habitats of *Panax notoginseng* under climate change based on MaxEnt and chemometric methods

**DOI:** 10.1038/s41598-024-67178-4

**Published:** 2024-07-16

**Authors:** Yixin Guo, Shiyan Zhang, Linghui Ren, Xin Tian, Shicheng Tang, Yisha Xian, Xinjia Wu, Zilong Zhang

**Affiliations:** 1 School of Chinese Materia Medica, Beijing University of Chinese Medicine, Beijing, 102488 China; 2 Meteorological Administration Key Open Laboratory of Transforming Climate Resource to Economy, Chongqing, 401147 China

**Keywords:** *P. notoginseng*, MaxEnt, Quality regionalization, Ecological regionalization, Climate change, Prediction model, Ecology, Plant sciences, Climate sciences, Ecology

## Abstract

Notoginseng saponin R1; ginsenosides Rg1, Re, Rb1, and Rd; the sum of the five saponins; and underground-part fresh weight (UPFW) of single plants were used as quality evaluation indices for *Panax notoginseng* (Burk.) F. H. Chen (*P. notoginseng*). Comprehensive evaluation of *P. notoginseng* samples from 30 production areas was performed using that MaxEnt model. Spatial pattern changes in suitable *P. notoginseng* habitats were predicted for current and future periods (2050s, 2070s, and 2090s) using SSP126 and SSP585 models. The results revealed that temperature, precipitation, and solar radiation were important environmental variables. Suitable habitats were located mainly in Yunnan, Guizhou, and Sichuan Provinces. The distribution core of *P. notoginseng* is predicted to shift southeast in the future. The saponin content decreased from the southeast to the northwest of Yunnan Province, which was contrary to the UPFW trend. This study provides the necessary information for the protection and sustainable utilization of *P. notoginseng* resources, and a theoretical reference for its application in the quality evaluation of Chinese medicinal products.

## Introduction

*Panax notoginseng* (Burk.) F. H. Chen is a perennial herb of the *Panax* genus (Araliaceae), whose medicinal parts are dry roots and rhizomes^1^. *P. notoginseng* is distributed in Yunnan Province, Guizhou Province, Guangxi Province, Chongqing City, and other regions^2^. It disperses blood stasis, reduces swelling, and relieves pain. Blood circulation promotion is the main function of saponins, including notoginseng saponin R1 and ginsenosides Rg1, Re, Rb1, and Rd^3^. Presently, more than 100 saponins have been described in *P. notoginseng* and are often used to treat hematemesis (vomiting blood), traumatic bleeding, and chest and abdominal (tingling pain)^4^. The medicinal components of *P. notoginseng* are found under the ground. Underground-part fresh weight (UPFW) is related to Notoginseng Radix et Rhizoma yield. Therefore, the saponin content and UPFW reflect the Notoginseng Radix et Rhizoma quality, and yield, respectively. These findings are of importance in evaluating the quality of Notoginseng Radix et Rhizoma.

Since ancient times, medicinal plants distribution in China has changed. Climate change majorly affects on the distribution of medicinal plants. For example, the upslope distribution of many mountain biological communities has changed owing to climate warming, which was also one of the reasons for the changes in Eucommia ulmoides distribution^5^. The primary and secondary metabolic pathways of medicinal plants are affected by climate change. The active components in most herbs are derived from secondary metabolic processes. Therefore, studying the environmental impacts of climate change on medicinal plant distribution and quality is conducive to sustainable development.

*P. notoginseng* has continuous cropping obstacles in the planting process, as it needs to grow for at least 3 years to reach the standard composition for medicine, and the land needs to be fallow^6^. Recently, the demand for Chinese medicinal materials has increased, leading to the short supply of *P. notoginseng* in markets. *P. notoginseng* from different areas have inconsistent product quality, which is not beneficial to standardized quality management of this Chinese medicinal material. Additionally, global warming may affect the distribution, saponin content, and UPFW of *P. notoginseng*.

MaxEnt model can predict potentially suitable habitats for *P. notoginseng* and provide guidance for expanding the planting areas^7,8^. The objective determination of quality differences between the regions is necessary to evaluate the quality of *P. notoginseng* in different regions^9^. To our knowledge, this is the first study to predict the current and future potentially suitable habitats of *P. notoginseng* and analyze changes in its suitable habitats under climate change. The correlation between *P. notoginseng* saponins, UPFW, and environmental variables was analyzed to identify limiting factors and establish a model^10^. The results were then superimposed on an ecological regionalization map to obtain quality distribution patterns. This is beneficial in identifying the distribution of indicators in various regions and has a certain guiding significance for scientific research and production.

## Materials and methods

### *P. notoginseng* distribution data

**Figure 1:**
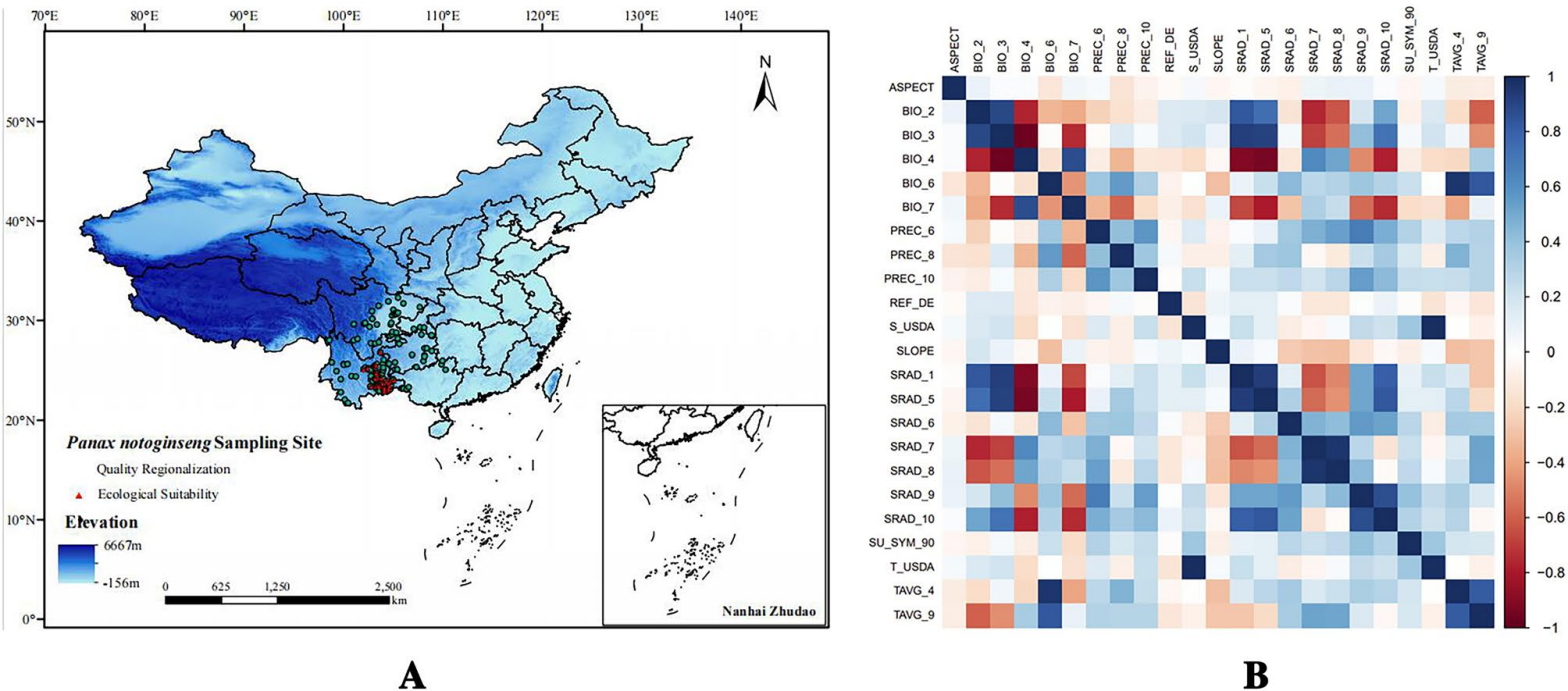
Pictures of (**A**) *Panax notoginseng* distribution in China. (**B**) Correlation heat map of environment variables.

Using field sampling and collecting data from the Global Biodiversity Information Facility (https://www.gbif.org/), Chinese Virtual Herbarium (http://www.cvh.ac.cn/), National Specimen Information Infrastructure (http://www.nsii.org.cn), the Plant Photo Bank of China (http://ppbc.iplant.cn), and previous studies, 201 occurrence records of *P. notoginseng* were collated. These were mainly distributed in southwest China, including Yunnan, Guangxi, Sichuan, and Guizhou Provinces^11,12^. The collected samples were from 30 producing areas in 18 regions distributed across southeastern Yunnan Province (Suppl. Material Table S1). To reduce sampling bias, only one record per grid (1 × 1 km) was selected^13^. Therefore, after removing the distribution points of longitude and latitude duplications and errors, 133 species distribution records that met the conditions were obtained using a buffer analysis method and saved in the CSV format available for the MaxEnt model (Fig. 1A).

### Handling of environmental variables

Current climate data were used as the baseline data (1970–2000), including 19 climate variables and 12-month averages for temperatures, solar radiations, and precipitations^14^. The future periods used for climate data were 2050s, 2070s, and 2090s. These climate data were selected from the Beijing Climate Center-Climate System Model-Medium Reslution (BCC-CSM2-MR) (Coupled Model Intercomparison Project Phase 6, CMIP6), which has two shared socioeconomic pathways (SSP126 and SSP585)^13,15^. These data were obtained from the WorldClim database (http://www.worldclim.org/) at a data spatial resolution of 2.5 m (1 × 1 km).

The elevation, slope, and aspect were also obtained from the WorldClim database (http://www.worldclim.org/) at the same spatial resolution. Thirty-six soil variables were obtained from the Harmonized World Soil Database (http://www.fao.org/soil s-portal/soil-survey/soil-maps-and-database s/harmonized-world-soil-database -v12/en/)^16,17^. Therefore, 94 environmental variables (Suppl. Material Table S2) were used in the model analysis for the current period. Terrain, soil, and climate data were not available for future scenarios. ArcGIS was used to extract and transform the data into the format required for the MaxEnt model operation^18^.

Environmental variables with percentage contribution > 0 were filtered out. When the Pearson correlation between two variables was > 0.80, the lower contribution rate was eliminated, and 10 environmental factors were selected to be used in the model analysis.

### Ecological regionalization model establishment

#### Prediction model and parameter optimization process

Almost 75% *P. notoginseng* distribution points were randomly selected as the training set to establish the prediction model, and the remaining 25% of the distribution points were selected as the test set to verify the model^19^. The jackknife method was selected to measure the importance of each variable, and the response curves of the environmental variables were created and projected onto the future environmental situation. This specific parameter setting was referred to as the optimized value, and the operation was repeated 10 times^20,21^.

In the MaxEnt model default values, the regularization multiplier (RM) was 1. Maxent has five features: linear (L), quadratic (Q), product (P), threshold (T), and hinge fragmentation (H)^22^. Model parameter optimization can avoid overfitting to a certain extent^23^. Delta Akaike information criterion coefficient (DELTA AICc) and omission rate (OR) were used to evaluate the degree of model optimization, using RGui scripts to simulate the different combinations of conditions. DELTA AICc, a standard measure of the goodness-of-fit of a statistical model, is calculated using R software. It can weigh the model complexity and fit the goodness-of-fit of the data, prioritizing the model with the lowest DELTA AICc. OR refers to the percentage of abnormal samples in the test sample that are not correctly classified to the total number of abnormal samples in the test sample; a small OR indicates a high prediction accuracy. The OR provides information on model differences and overfitting and evaluates the data used at a specific threshold. The model parameters were optimal when the OR was < 5% and the DELTA AICc was minimal^24,25^.

#### Classification of suitable habitual levels and changes in core distribution

The reclassification tool ArcGIS was used to classify the ecological suitability of *P. notoginseng*. Suitable habitats were divided into four levels: unsuitable (0–0.1), poorly suitable (0.1–0.25), moderately suitable (0.25–0.5), and highly suitable (0.5–1)^13^. Four different colors were used to distinguish the habitats of each level.

The SDM Toolbox v2.4 in ArcGIS converted the MaxEnt operation results into binary, converted suitable habitats at different periods into particles, and then analyzed their movement trajectory and distance^26^.

### Quality regionalization analysis

#### Saponin analysis

For quality analysis, samples from 3-year-old *P. notoginseng* were collected from 30 sampling sites (Fig. 1A). Notoginseng saponin R1 and ginsenosides Rg1, Re, Rb1, and Rd (National Institutes for Food and Drug Control, Beijing, China) were prepared as standard solutions and five test saponin solutions were prepared as reported by Chinese Pharmacopoeia, 2020 edition^1^ and Zhang et al.^27^. Five saponin solutions were assessed using an e2695 high-performance liquid chromatograph (Waters Corporation, MA, USA). The chromatographic column was an XBridge C_18_ column (250 × 4.6 mm, 5 μm). The mobile phase was acetonitrile–water gradient elution using 0–20 min, 20% acetonitrile; 20–45 min, 20–45% acetonitrile; 45–55 min, 45–55% acetonitrile; 55–60 min, 55% acetonitrile; and 60–70 min, 55–20% acetonitrile. The flow rate, detection wavelength, sample size, and column temperature were 1 mL·min^−1^, 203 nm, 20 µL, and 25 ℃, respectively. The sum of the five saponins peaks was the total saponin content^27–30^. Subsequently, the linear relationship and methodology were investigated. The chromatograms of mixed reference and *P. notoginseng* test solutions under these conditions are presented in Suppl. Material Fig. S1.

*P. notoginseng* saponins (PNS) were determined as reported by Qin et al.^31^ and Liu^29^. A UV 2800 ultraviolet–visible spectrophotometer (Sunny Hengping Instrument, Shanghai, China) was used to scan 190–700 nm, and a maximum absorption wavelength of 546 nm was selected as the determination wavelength. The linear relationship has previously been investigated^32,33^.

Correlation and stepwise regression analyses were carried out on the data from notoginseng saponin R1; ginsenosides Rg1, Re, Rb1, and Rd; and the sum of five saponins in terms of PNS content and environmental factors. Models for chemical constituents and environmental factors were established. The kriging spatial interpolation method was used to construct the model when no regression equation was established for the environmental variables^34^. The chemical composition distribution map obtained by constructing the model method and the ecological suitability habitat map after reclassification were superimposed to obtain a quality regionalization map^30^.

#### Analysis of UPFW of a single plant

The UPFW of *P. notoginseng* samples from each sampling site was measured using an electronic scale with 0.01 g accuracy and average values were taken from each sampling site for analysis. The UPFW data and environmental factors were analyzed using stepwise regression, a model for UPFW, and environmental factors was established. The kriging spatial interpolation method was used to construct the model when no regression equation was established for the environmental variables. The UPFW distribution map obtained by constructing the model method and the ecological suitability habitat map after reclassification were superimposed to obtain a quality regionalization map^35^.

## Results

### Credibility evaluation

The predictive accuracy of the MaxEnt model was measured using the area under curve (AUC)^36^. The AUC value was generally 0–1, in which 0–0.5 indicate model prediction failure. AUC 0.6–0.7, 0.7–0.8, 0.8–0.9, and > 0.9 indicate poor, general, good, and excellent prediction effect, respectively^37^. In the current period, 1,160 candidate models were generated after parameter optimization, with 193 and 3 models meeting the OR criterion and AICc, respectively. However, only one statistically significant model met both the OR and AICc. The parameters of this model were set as follows: FC = LPT and RM = 2.4. The DELTA AICc of this model was 0 and AUC value 0.965 ± 0.005 (Suppl. Material Fig. S2). For the future period, a total of 1160 candidate models were generated after parameter optimization, with 400 and 1 models meeting the OR criterion and AICc, respectively. However, only one statistically significant model met the OR criterion and AICc. The parameters for this model were set at FC = LP and RM = 0.1. The DELTA AICc of this model was 0 and the AUC value was 0.9640 ± 0.008 (Suppl. Material Fig S3). This result indicates high model fitting effect and reliable experimental results.

### Environmental variable analysis

The environmental variables were filtered as described in Section “Handling of environmental variables”, and 14 environmental variables were obtained for modeling. A heat map of the correlations between these parameters was generated (Fig. 1B and Suppl. Material Table S3). The permutation importance and percentage contribution of the replacement of the 14 environmental variables in the model were compiled (Suppl. Material Table S4). In summary, BIO_7, SRAD_6, SRAD_7, SRAD_10, and PREC_8 were the key factors limiting *P. notoginseng* growth and development and their permutation importance and percentage contributions were 90.1 and 89.6, respectively.

**Figure 2:**
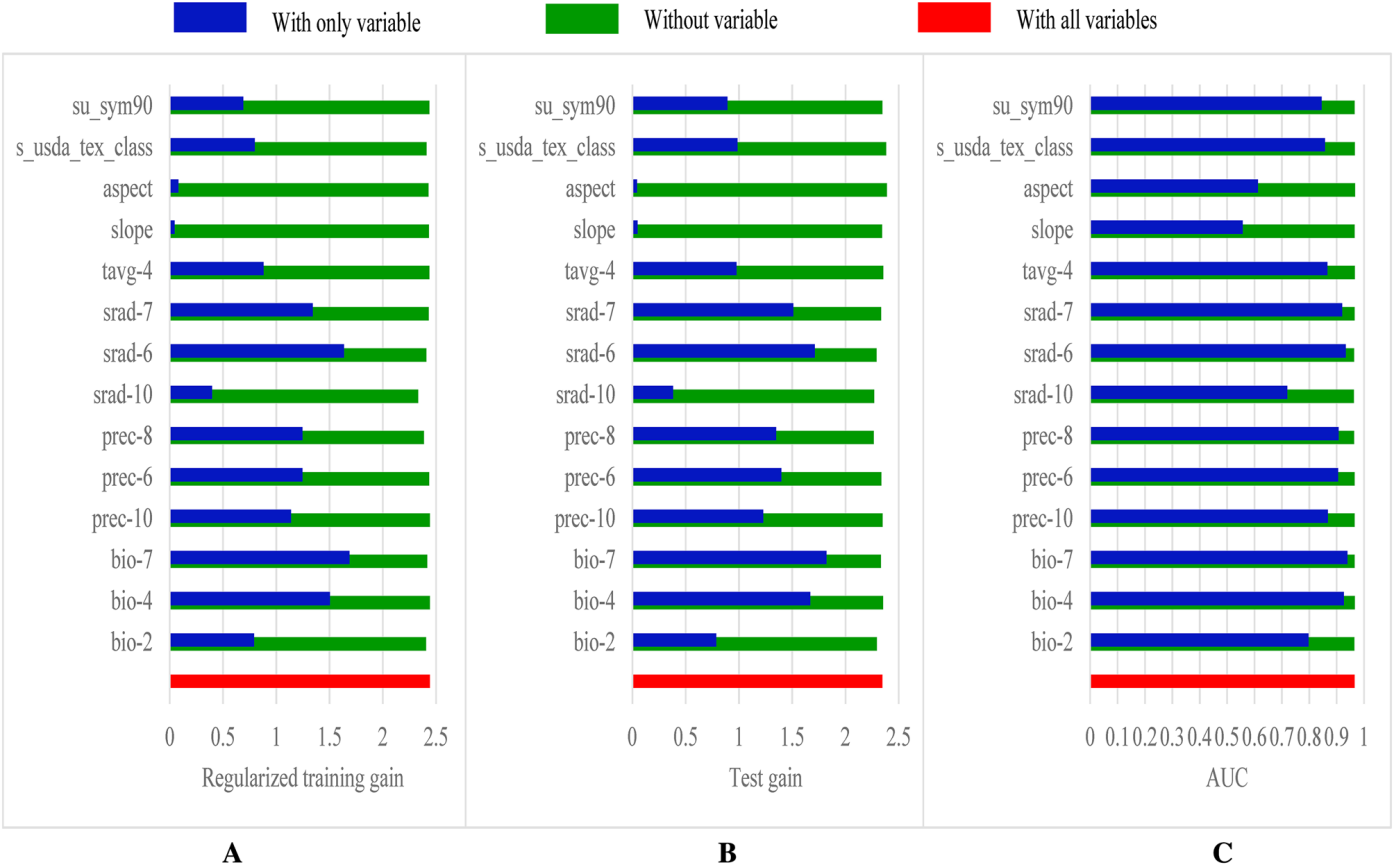
Jackknife results of (**A**) regularized training gain for *P. notoginseng*, (**B**) test gain, and (**C**) AUC for *Panax notoginseng.*

According to the jackknife test results, the environmental variables of temperature, precipitation, and solar radiation had greater than 1.1 regularization training and test gain values, with AUC > 0.9 (Fig. 2). These results suggest that environmental variables affect the geographical distribution of medicinal plants.

### Potential suitable habitats and optimal environmental conditions of *P. notoginseng* in the current period

**Figure 3:**
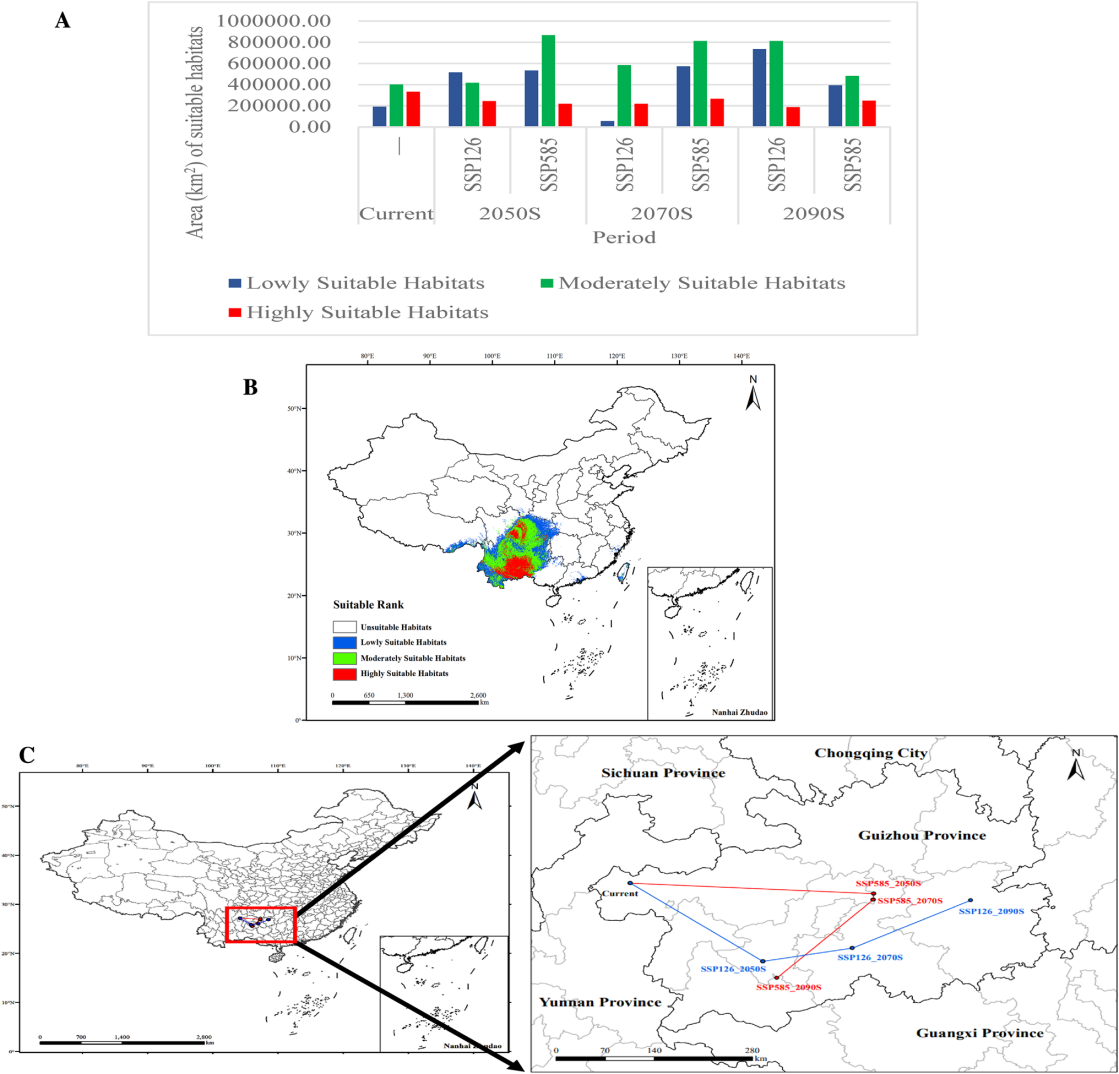
Changes in the distribution of *Pananx notoginseng*. (**A**) Area of *P. notoginseng* suitable habitats under different periods and climate scenarios. (**B**) Ecological regionalization distribution of *P. notoginseng* in current period. (**C**) Centroid displacement of *P. notoginseng* ecological regionalization.

In the current period, the total suitable habitat area for *P. notoginseng* in China is 924,676.79 km^2^ (Fig. 3A). Among these, the area of highly suitable habitats was 332,953.01 km^2^, distributed in the southeastern Yunnan Province, southern Guizhou Province and eastern Sichuan Province. The area of moderately suitable habitats was 400,844.21 km^2^, distributed in central and western Yunnan Province, most of Guizhou Province, northwestern Guangxi Province, southeastern Sichuan Province, and Chongqing City. The area of poorly suitable habitats was 190,879.57 km^2^, distributed in the area surrounding moderately suitable habitats. Moderately and poorly suitable habitats surrounded and radiated around highly suitable habitats (Fig. 3B). This is consistent with the results reported by Zhan et al.^13^.

According to the response curve results of environmental variables, *P. notoginseng* growth was the most advantageous with 10–15 ℃ annual temperature range; 500–600 mm average precipitation in August; and 1500–1600, 18,500–19,500, and 8000–9000 kJ·m^−2^ day^−1^, average solar radiation in June, July, and October, respectively.

### Changes of *P. notoginseng* distribution in the future

#### Changes of *P. notoginseng* suitable habitats in the future

**Figure 4:**
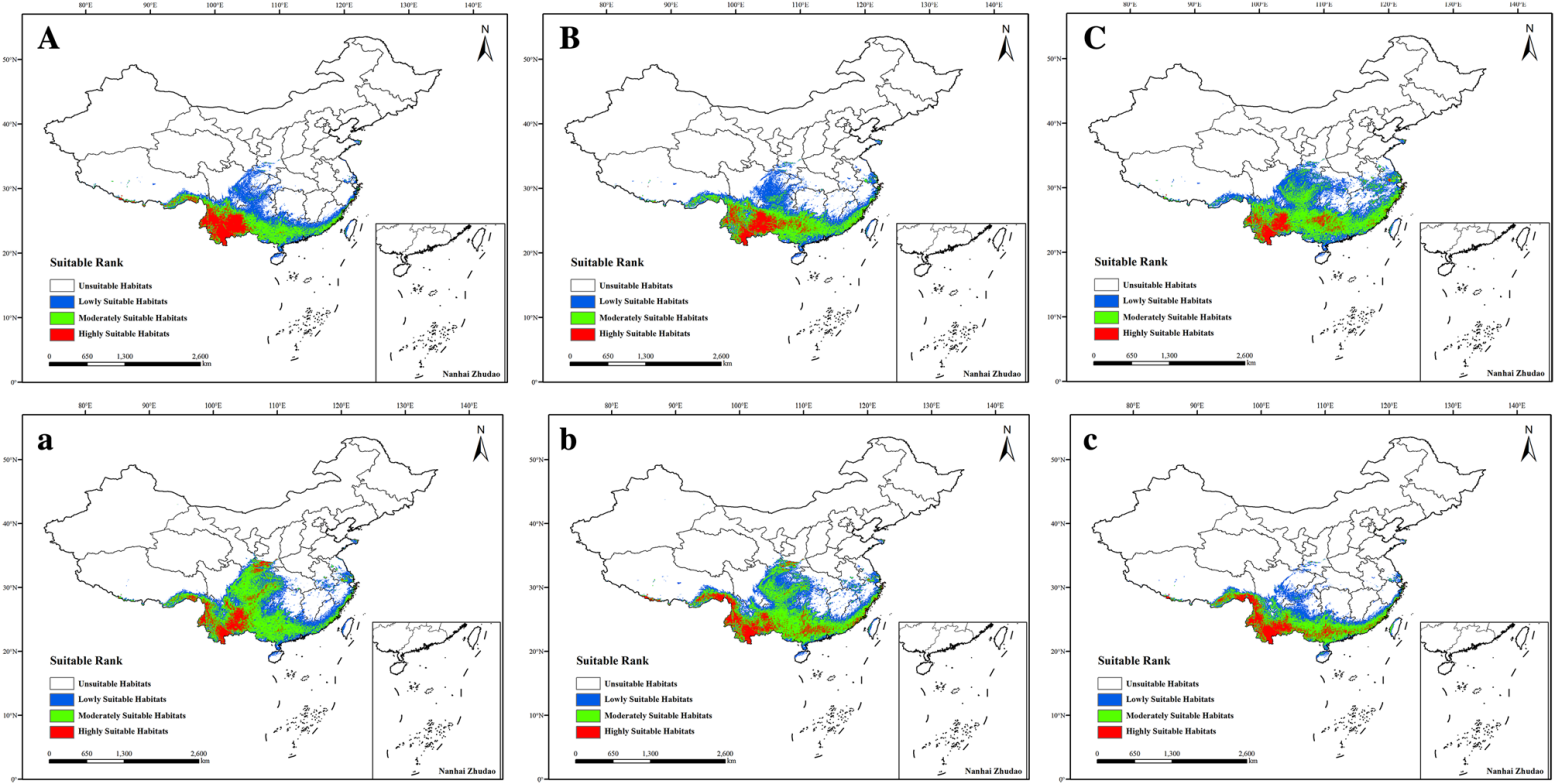
Suitable habitats for *Panax notoginseng* under different future climate scenarios. 2050s, 2070s, and 2090s using SSP126 (**A**–**C**, respectively); and 2050s, 2070s, and 2090s using SSP585 (a–c, respectively).

Examination of suitable *P. notoginseng* habitat distribution in different future periods and climate scenario models revealed that the trend of the total area of suitable habitats was to first expand, then reduce, and then expand in the SSP126 model (Fig. 4). Among these, the largest suitable habitats appeared in 2070s (1,648,892.29 km^2^), which might be because the increase in area of moderately and highly suitable habitats of *P. notoginseng* in this period. The trend of the total area of suitable habitats first expanded and then reduced in the SSP585 model. The largest suitable habitats appeared in 2090s in the SSP126 model (1,732,954.80 km^2^), which might be owing to relatively balanced proportion of poor, moderate, and highly suitable habitats of *P. notoginseng* in this period. The changes in area for each level of suitable habitat is presented in Fig. 3A. Changes in the distribution, expansion, shrinkage, and stability of ecological regionalization in future periods and climate scenarios were compared with those in the current period (Table 1).

**Table 1:** Stable, shrunken, and increased areas (km^2^) of suitable habitat for *Panax notoginseng* in the future.

	2050S		2070S		2090S	
	SSP126	SSP585	SSP126	SSP585	SSP126	SSP585
Extented area (km^2^)	542,119.71	814,712.44	688,141.41	916,242.34	975,496.03	591,119.94
Stable area (km^2^)	777,640.24	982,761.05	822,879.14	917,581.99	953,366.06	671,060.89
Shrink area (km^2^)	254,713.57	49,592.76	209,474.67	114,771.82	78,987.74	361,292.92

#### Change of *P. notoginseng* centroid displacement

During the present period, the distribution core was in Weining County, Bijie City, Guizhou Province (104.14146° E, 27.157767° N). According to the SSP126 model, by 2050s the distribution core migrated 224.30059 km southeast to Zhenning County, Anshun City, Guizhou Province (105.840825° E, 25.830653° N). In 2070s, it migrated 117.26312 km northeast to Huishui County, Qiannan Prefecture, Guizhou Province (106.988205° E, 26.051955° N). In 2090s, it continued to migrate 176.03582 km northeast to Zhenyuan County, Qiandongnan Prefecture, Guizhou Province (108.50604° E, 26.866239° N). According to the SSP585 model, by 2050s the distribution core migrated 309.36487 km southeast to Fuquan City, Qiannan Prefecture, Guizhou Province (107.261263° E, 26.976562° N). In the 2070s, it migrated 11.39977 km south to Kaiyang County, Guiyang, and Guizhou (107.255406° E, 26.87411° N). In 2090s, it migrated 192.05186 km southwest to Wangmo County, Qianxinan Prefecture, Guizhou (106.022909° E, 25.545873° N). In the two climate scenarios, the centroid displacement trends were opposite although in the same direction (Fig. 3C).

### Prediction of changes in *P. notoginseng* saponins

#### Saponin content in *P. notoginseng*

Analyses of 300 samples from 30 production areas are presented in Suppl. Material Fig. S4. The concentration of notoginseng saponin R1 content in 179 samples was 0.5–1.5%. The concentration of ginsenoside Rg1 content in 127 samples was 2.5–4.0%. The ginsenoside Re content in 142 samples was < 0.5%. The concentration of ginsenoside Rd content in 140 samples was 0.5–1.0%. The sum of five saponin content in samples was 7.5–11.5%. The concentration of PNS content in 122 samples was 6.0–10.5%.

#### Construction of a regression model

The content of notoginseng saponin R1; ginsenosides Rg1, Re, Rb1, and Rd; the sum of five saponins; and PNS from 30 regions were measured, and stepwise regression analysis was performed with environmental variables to obtain the corresponding relationship model.

The relationship model between notoginseng saponin R1 and environmental variables was:

1 $$ {\text{Y}}_{{1}} = { 1}.{7}0{9 }{-} \, 0.0{\text{73 X}}_{{1}} + \, 0.0 43{\text{X}}_{{2}} , $$

where Y_1_ is the notoginseng saponin R1 content; X_1_ is the average precipitation in October, and X_2_ is the average precipitation in June. In the regression equation F-test, *P* = 0.012 (< 0.05), F = 1.026, and R^2^ = 0.280, indicating the significant effect of the equation.

The relationship model between ginsenoside Rg1 and environmental variables was:

2 $$ \begin{aligned} & {\text{Y}}_{{2}} = \, - {37}.{878 } + \, 0.{\text{585 X}}_{{3}} + \, 0.0{\text{4 X}}_{{4}} {-} \, 0.{\text{353X}}_{{5}} {-} \, 0.{\text{21 X}}_{{1}} + \, 0.0{\text{67 X}}_{{2}} + \, 0.0{\text{26 X}}_{{6}} {-} \, 0.00{1} \\ & {\text{X}}_{{7}} + \, 0.00{\text{5 X}}_{{8}} {-} \, 0.00{\text{2 X}}_{{9}} {-} \, 0.0{\text{2 X}}_{{{1}0}} + \, 0.{\text{14 X}}_{{{11}}} , \\ \end{aligned} $$

where Y_2_ is the Rg1 content; X_1_ is the average precipitation in October; X_2_ is the average precipitation in June; X_3_ is the mean diurnal range; X_4_ is the temperature seasonality; X_5_ is the annual temperature range; X_6_ is the average precipitation in August; X_7_, X_8_, and X_9_ are the average solar radiation in October, June, and July, respectively; X_10_ is the average temperature in April; and X_11_ is the slope. In the regression equation F-test, *P* = 0.033 (< 0.05), F = 2.631, and R^2^ = 0.617, indicating the significant effect of the equation.

The relationship model between ginsenoside Rb1 and environmental variables was:

3 $$ \begin{aligned} {\text{Y}}_{{3}} & = \, - {148}.{751 } + { 3}.{\text{214 X}}_{{3}} + \, 0.{\text{148 X}}_{{4}} {-}{ 1}.{\text{938X}}_{{5}} {-} \, 0.{\text{299 X}}_{{1}} + \, 0.{\text{151 X}}_{{2}} + \, 0.0{\text{14 X}}_{{6}} \\ & \quad {-} \, 0.00{\text{2 X}}_{{7}} + \, 0.0{\text{15 X}}_{{8}} {-} \, 0.00{\text{7 X}}_{{9}} - \, 0.{\text{639 X}}_{{{1}0}} + \, 0.{\text{495 X}}_{{{11}}} , \\ \end{aligned} $$

where Y_3_ is the Rb1 content; X_1_ is the average precipitation in October; X_2_ is the average precipitation in June; X_3_ is the mean diurnal range; X_4_ is the temperature seasonality; X_5_ is the annual temperature range; X_6_ is the average precipitation in August; X_7_, X_8_, and X_9_ are the average solar radiation in October, June, and July, respectively; X_10_ is the average temperature in April; and X_11_ is the slope. In the regression equation F-test, *P* = 0.027 (< 0.05), F = 2.770, and R^2^ = 0.629, indicating the significant effect of the equation.

The relationship model between ginsenoside Rd and environmental variables was

4 $$ {\text{Y}}_{{4}} = \, - {5}.{813 } + \, 0.0{\text{31 X}}_{{4}} {-} \, 0.{\text{456 X}}_{{{1}0}} + \, 0.{\text{842 X}}_{{{11}}} , $$

where Y_4_ is the Rd content, X_4_ is the temperature seasonality, X_10_ is the average temperature in April, and X_11_ is the slope. In the regression equation F-test, *P* = 0.000 (< 0.05), F = 15.396, and R^2^ = 0.640, indicating the significant effect of the equation.

The relationship model between the sum of five saponins and environmental variables was:

5 $$ {\text{Y}}_{{5}} = { 2}0.{736 } + \, 0.00{\text{2 X}}_{{4}} {-} \, 0.{3}0{\text{3 X}}_{{1}} + \, 0.0{\text{62 X}}_{{6}} , $$

where Y_5_ is the sum of the five saponins, X_1_ is the average precipitation in October, X_4_ is the temperature seasonality, and X_6_ is the average precipitation in August. In the regression equation F-test, *P* = 0.034 (< 0.05), F = 3.359, and R^2^ = 0.279, indicating that the effect of the significant equation.

The stepwise regression equation between ginsenoside Re, PNS content, and environmental variables was not significant. Hence, no regression equation was established.

#### Distribution analysis of *P. notoginseng* saponins

**Figure 5:**
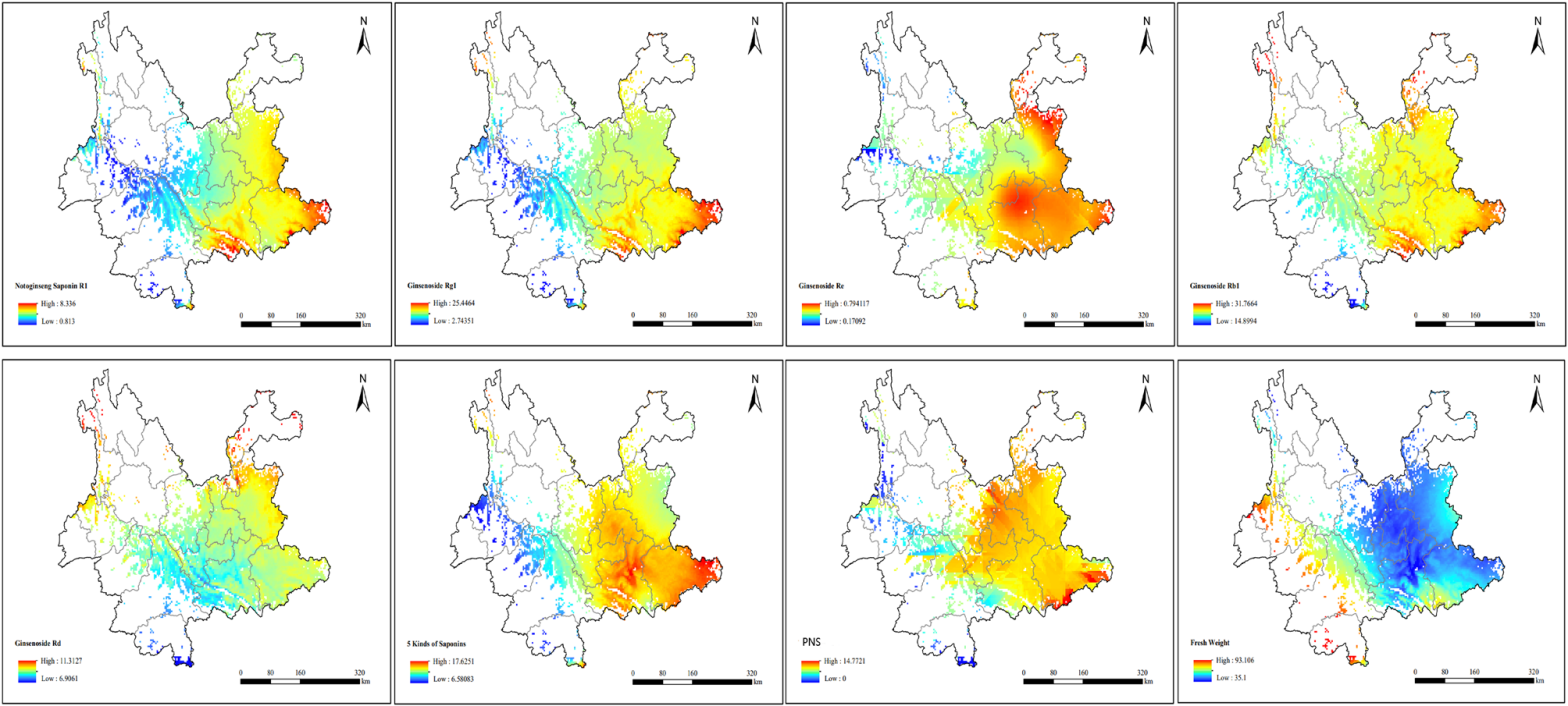
Spatial distribution of *Panax notoginseng* qualities*.*

Based on the regression equation established between saponin components and environmental variables as well as the kriging spatial interpolation method, ArcGIS was used for spatial calculations to obtain the spatial distribution of notoginseng saponin R and ginsenosides Rg1, Re, Rb1, and Rd; the sum of five saponins; and PNS content (Fig. 5).

Regions with high saponin components were found in lower altitudes. Regions with high levels of notoginseng saponin R1 and ginsenosides Rg1 and Rb1 were in the Wenshan and Honghe Prefectures of Yunnan Province, with similar trends of change, decreasing from the southeast to the northwest. The ginsenoside Re content was relatively high in Wenshan Prefecture, Honghe Prefecture, Kunming City, and Qujing City in Yunnan Province. The ginsenoside Rd content was generally low in most areas of Yunnan Province. There were certain differences in the spatial distribution of the sum of five saponins and PNS content, indicating that the sum of five saponins did not fully reflect the overall changes in saponin components in *P. notoginseng*.

### Spatial distribution analysis of UPFW of *P. notoginseng*

Using the measured UPFW from 30 regions and environmental variables for stepwise regression analysis, a corresponding relationship model was obtained. The relationship between fresh weight and environmental variables was as follows:

6 $$ {\text{Y}}_{{6}} \, = \,{3}.{4}0{6}\, + \,0.{\text{598 X}}_{{1}} , $$

where Y_6_ is the fresh weight and X_1_ is the average precipitation in October. In the regression equation F-test, *P* = 0.045 (< 0.05), F = 4.399, and R^2^ = 0.136, indicating the effect of the significant equation.

Based on the regression equation established between the UPFW and environmental variables, ArcGIS was used for spatial calculations and then overlaid with the ecological suitability habitat map to obtain a distribution map of the UPFW of *P. notoginseng* (Fig. 5).

Comparing the UPFW and PNS distribution maps demonstrated an inversely significant relationship between them. In Yunnan Province, the UPFW were lower in areas with low elevations as well as areas with high saponin content. Among them, Maguan, Wenshan, and Pingbian Prefectures in Honghe Prefecture had higher UPFW and saponin content, which could potentially increase *P. notoginseng* cultivation.

## Discussion

### Model precision analysis

Recently, machine learning-based modeling methods have been widely used to predict the ecological distribution of many species^38^. MaxEnt is one of the most widely used and recognized models^39^. It not only analyzes climate, soil, terrain and other environmental factors, but also considers the impact of human activities on the modeling process. When predicting the species distribution on a small scale, human factors such as vegetation cover and the urbanization index can be added to the model to help make the prediction more consistent with the actual situation. In this study, the control unit grating had only one species distribution point, overfitting environmental variables were removed, and the R software was used for parameter optimization. The AUC of the optimized model in both the training and test sets were > 0.9, which improved model accuracy and reliability.

### Migration of suitable habitats for *P. notoginseng* under climate change conditions

BCC-CSM2-MR has four modes: SSP126, SSP242, SSP370, and SSP585. Among them, there was a significant difference between SSP126 and SSP585, which was representative. Therefore, these two factors were chosen as the focus of this research^40^. The distribution of suitable habitats for *P. notoginseng* was most closely related to annual temperature range, solar radiation, and precipitation. *P. notoginseng* grows in dark and humid environments, which was confirmed from the environmental factors obtained by the model. Under the general trend of global warming, the minimum temperature will increase, solar radiation will enhance, and the future suitable habitats of *P. notoginseng* will thereby be greatly affected and experience change^41^.

The predicted potentially suitable habitat distribution of *P. notoginseng* in this study was different from that reported by Zhan et al.^13^. The number of species distribution data inputs in MaxEnt and the selection of environmental variables had an impact on the modeling effect and result output^42^. The ecological regionalization area in this study was larger than that of the previous study; however, the suitable habitat conditions of the covered areas was consistent. This was because the distribution of *P. notoginseng* in Chongqing City, and the overall collection range of the sampling points might have been more extensive in this study. The distribution of *P. notoginseng* in China is characterized by a subtropical monsoon climate. The rain and heat brought about by this climate are conducive to the growth and accumulation of effective components of *P. notoginseng*^43^. The southeastern part of Yunnan Province and the southern part of Guizhou Province belong to the plateau, that is, the Yungui Plateau. Ocean currents from the Pacific Ocean can directly affect this area, resulting in hot and humid climates, that are the best growth areas for *P. notoginseng*. However, western Yunnan and northern Guizhou are blocked by plateaus and mountains; the influence of ocean currents is greatly reduced, thereby lowering the fitness index of *P. notoginseng*. Although the genuine areas of *P. notoginseng* were Yunnan and Guangxi Provinces, a change in the distribution core occurred in Guizhou Province. This is because the position of the centroid was obtained by a binary operation on suitable habitats rather than on highly suitable habitats^44^. Therefore, the centroid displacement reflected changes in the overall suitable habitats of *P. notoginseng* during different periods, which was also more consistent with reality.

According to ancient Chinese books, *Panax notoginseng* was originally cultivated in Guangxi Province and later moved to Yunnan Province. During the Qing Dynasty, Notoginseng Radix et Rhizoma from Guangxi and Sichuan Provinces were presented to the emperor; however, researchers did not find any records of *P. notoginseng* cultivation in Sichuan Province. Researchers will continue to expand the collection of historical distribution and climate records of *P. notoginseng* in China, which will be of great importance for studying *P. notoginseng* distribution.

### Correlation analysis of saponin content and UPFW with environmental factors

**Figure 6:**
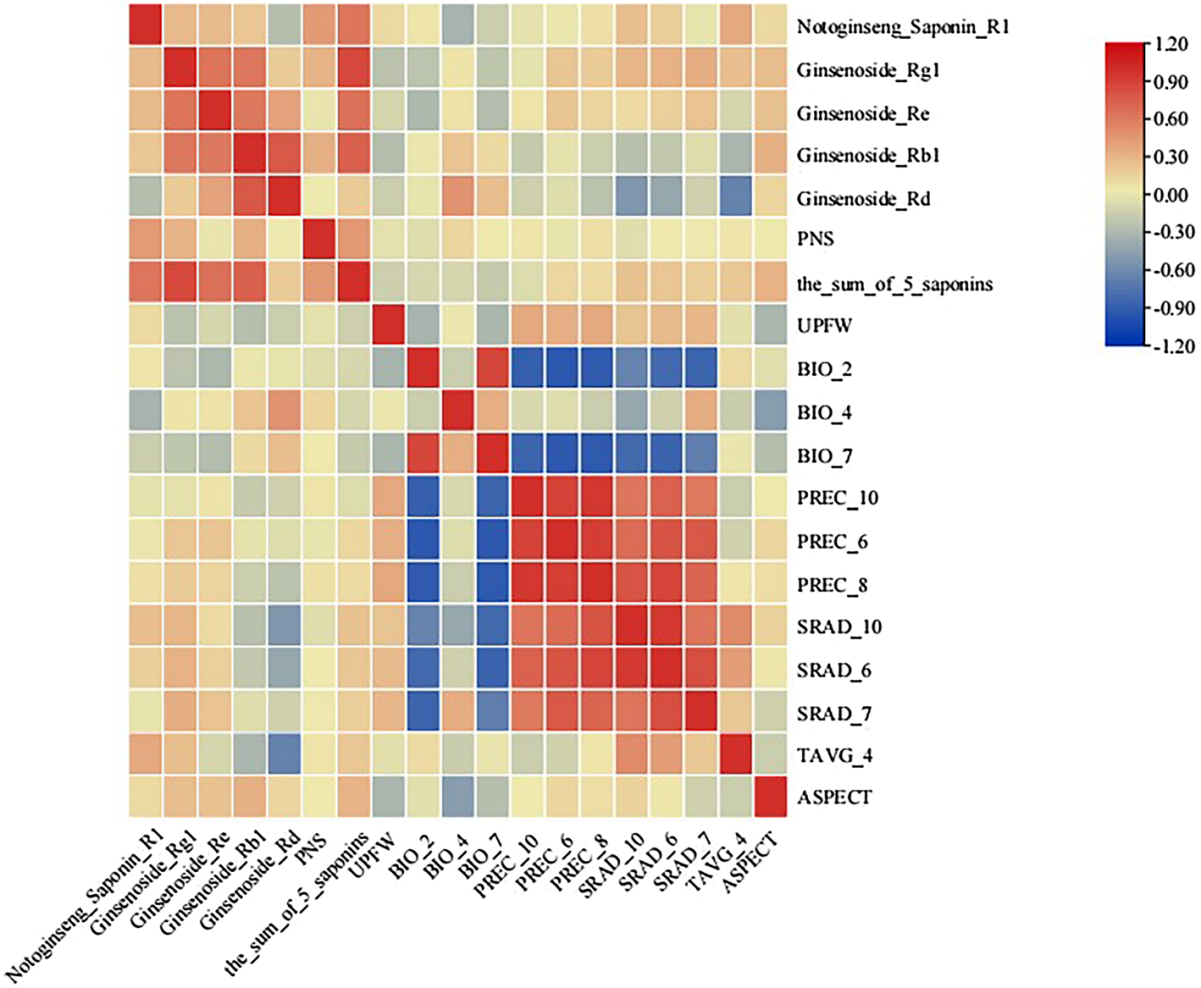
Correlation heat map of environment variables and Index of Notoginseng Radix et Rhizoma.

In the quality regionalization analysis of *P. notoginseng* saponins and UPFW, the prediction scope was narrowed to Yunnan Province as the 37 sampling points were concentrated in Yunnan Province. After correlating the quality and environmental variable data of Yunnan Province, the quality regionalization prediction was more representative and accurate. TB Tools v1.115 is used to draw correlation heat maps. The correlation analysis of environmental factors and quality indices revealed that notoginseng saponin R1 was significantly positively correlated with TAVG_4 (Fig. 6). Ginsenoside Rd positively correlated with temperature seasonality and negatively correlated with SRAD_10 and TAVG_4. UPFW was significantly and positively correlated with PREC_10 and PREC_8. Since the sampling points were concentrated in eastern Yunnan Province, quality regionalization could only predict changing trends. Owing to sampling point distribution and numbers, *P. notoginseng* quality regionalization of could be further improved. Although we attempted to use multiple models for joint predictions, these shortcomings could not be ignored. In addition, saponins are the main active component of *P. notoginseng* and play an important role in the treatment of trauma and cardiovascular and cerebrovascular diseases. In this study, the content of five saponins changed with the environment, which laid a foundation for the study of *P. notoginseng* quality and human health under the climate change.

In this study, we predicted the potential distribution area and future changes of *P. notoginseng* to a certain extent, which has guiding significance for expanding its cultivation in China. The ecological regionalization was innovatively combined with chemical composition and UPFW analysis, to intuitively present the change trend, which has a certain reference value in clinical medicine and the development of precise Chinese medicine.

## Conclusions

In this study, the MaxEnt model with optimized parameters was used to evaluate and predict the suitable habitat distribution for *P. notoginseng*. Based on the analysis results of the current period, the optimal *P. notoginseng* growth conditions were 10–15 ℃ annual temperature range; 500–600 mm average precipitation in August; and 1500–1600, 18,500–19,500, and 8000–9000 kJ·m^−2^·day^−1^ average solar radiation in June, July, and October, respectively. Highly suitable habitats were mainly distributed in the central and eastern Yunnan Province, southwest Guizhou Province, and Chongqing City. Other suitable habitats radiated around them and were distributed throughout southwestern China. In the future, owing to climate change conditions, *P. notoginseng* distribution core could shift to the southeast. Based on the analyses of saponin composition and single plant UPFW, saponin content decreased from southeastern to northwestern Yunnan Province and was the highest in Honghe and Wenshan Prefectures, Yunnan Province. The UPFW was inversely related to saponin content. The results of this study provide a reference for the planting status and prediction of suitable habitats for *P. notoginseng*. They lay a solid foundation and practical guidance for promoting *P. notoginseng* yield and quality.

### Supplementary Information

Supplementary Information.

## Data Availability

All data generated or analysed during this study are included in this published article. Experimental research and feld studies on plants (either cultivated or wild), including the collection of plant material are complying with relevant institutional, national, and international guidelines and legislation. I declare that the plant material used in our study was harvested from farmland. We did not use endangered plant species for the experiments.
