# Supplementary material for: Prediction of Chinese suitable habitats of Panax notoginseng under climate change based on MaxEnt and chemometric methods
Source: Sci Rep. 2024 Jul 16;14:16434. doi: 10.1038/s41598-024-67178-4 (PMC11252130; doi:10.1038/s41598-024-67178-4)
Supplement: Supplementary file 1 — Supplementary Information. [file 41598_2024_67178_MOESM1_ESM.docx]

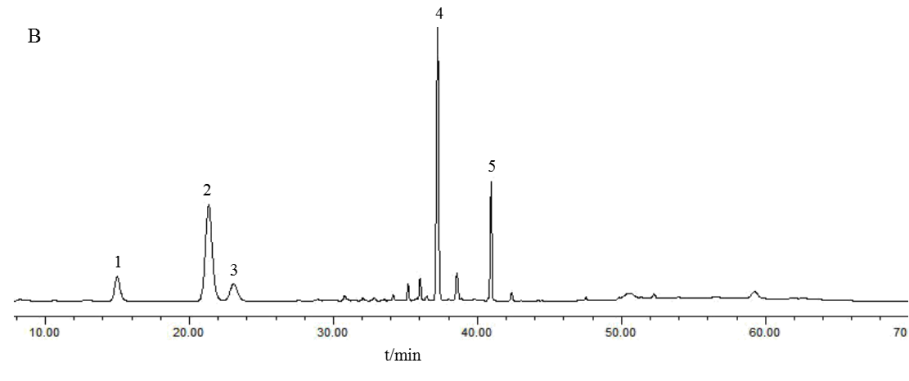

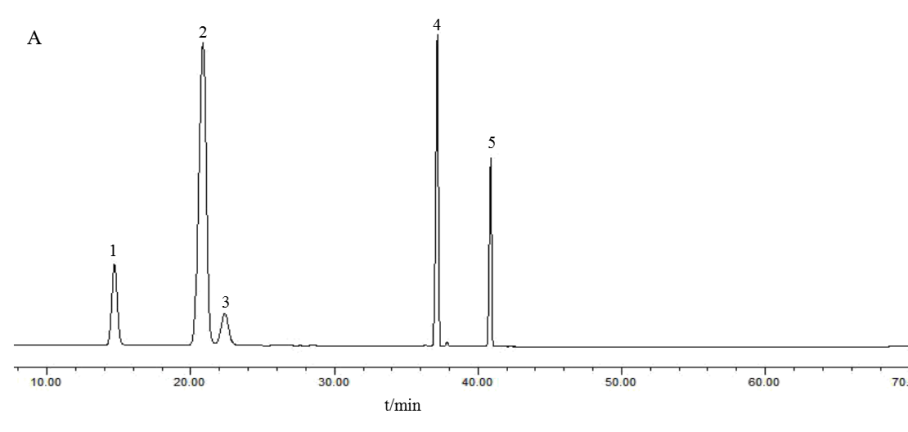
**Supplementary Material**

**Fig. S1 HPLC chromatograms of mixed standard sample (A) and test substance (B)**


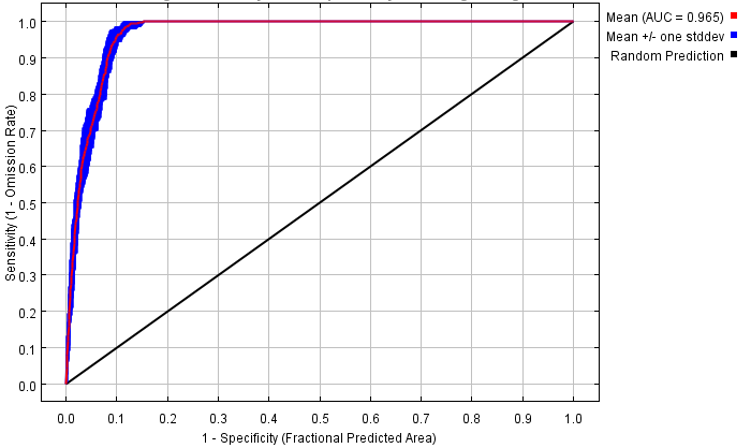


**Fig. S2 ROC curve of *P. notoginseng* prediction model under current climate conditions**


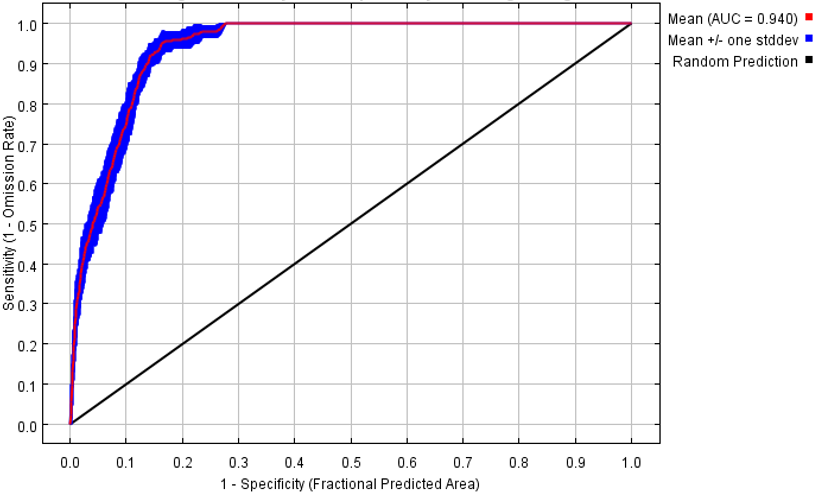
**Fig. S3 ROC curve of *P. notoginseng* prediction model under future climate conditions**


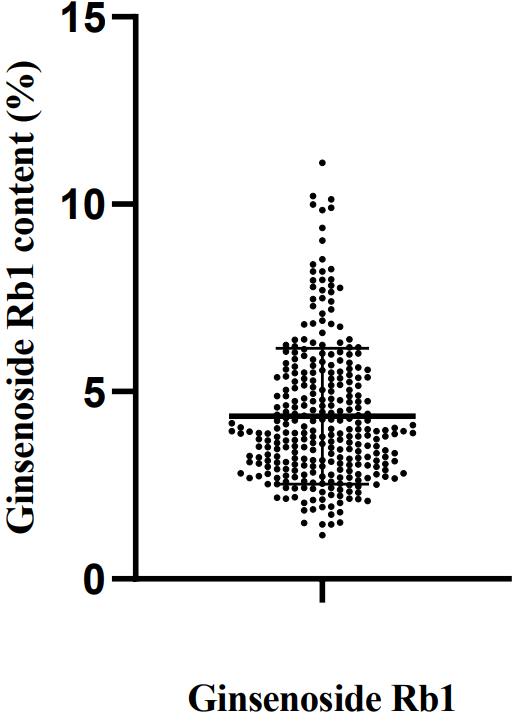

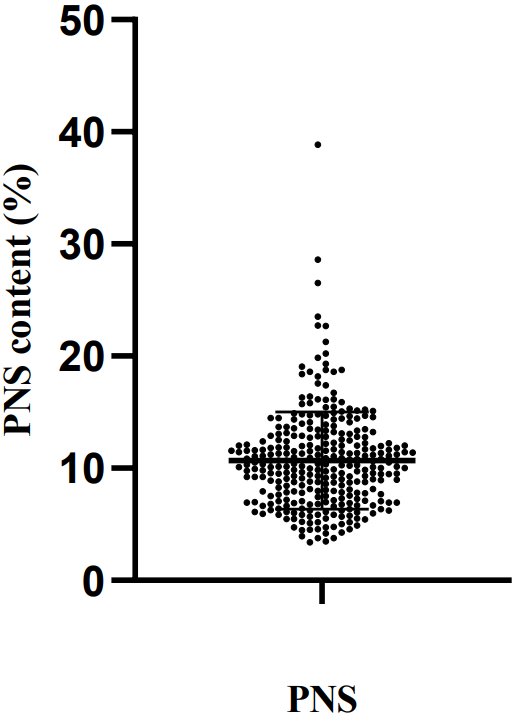

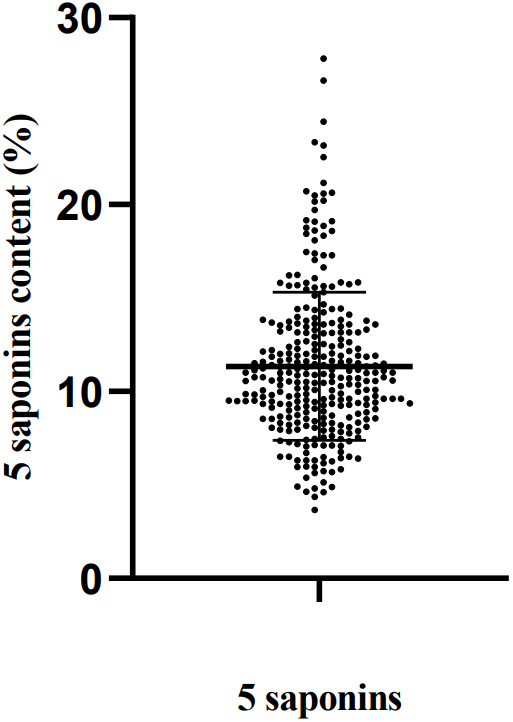

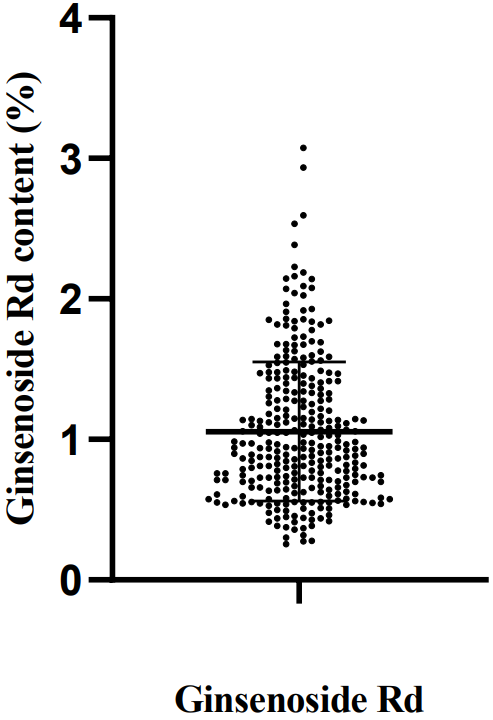

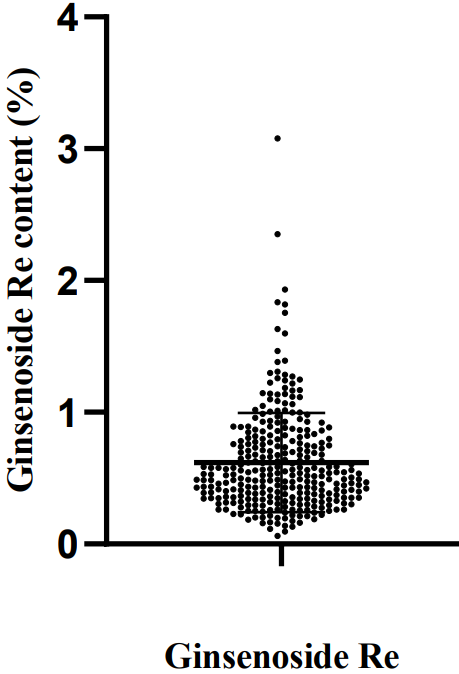

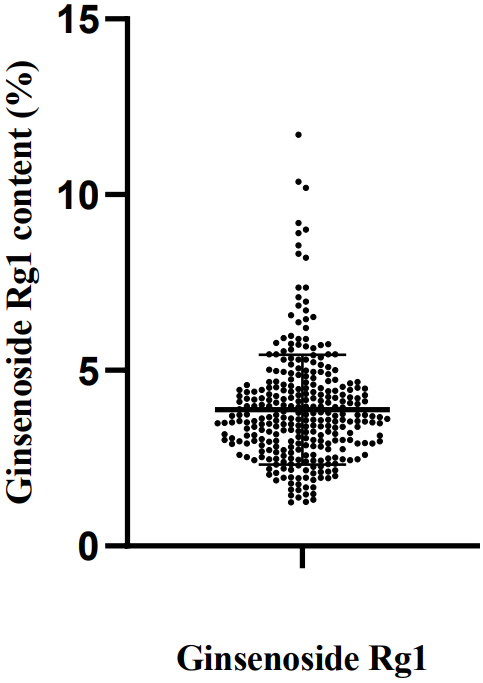

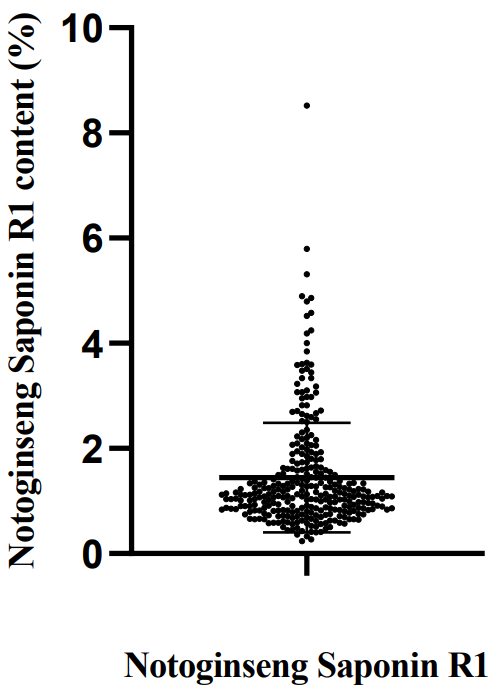


**Fig. S4. Scatter plot of saponin content distribution.**

**Table S1. Sampling point information of quality regionalization of *P. notoginseng***

| Origin | Longitude | Latitude |
| --- | --- | --- |
| CX-LF-1 | 102.0665 | 25.13574 |
| HH-GJ-1 | 103.2757 | 23.41026 |
| HH-JS-1 | 102.7233 | 23.40341 |
| HH-KY-1 | 103.517 | 23.56752 |
| HH-MZ-1 | 103.6498 | 23.44946 |
| HH-ML-1 | 103.453 | 24.24878 |
| HH-ML-2 | 103.2054 | 24.17022 |
| KM-FM-1 | 102.4953 | 25.22423 |
| KM-SL-1 | 103.4053 | 24.70634 |
| KM-SL-2 | 103.2932 | 24.78163 |
| KM-XD-1 | 103.1283 | 25.43809 |
| KM-XD-2 | 103.2111 | 25.51247 |
| QJ-LL-1 | 103.3327 | 25.53308 |
| WS-GN-1 | 105.0562 | 24.04792 |
| WS-GN-2 | 104.6703 | 23.93738 |
| WS-MG-1 | 104.3993 | 23.01382 |
| WS-MG-2 | 104.1853 | 22.93447 |
| WS-MLP-1 | 104.5919 | 23.10872 |
| WS-MLP-2 | 104.5895 | 23.10769 |
| WS-QB-1 | 104.5893 | 23.10772 |
| WS-QB-2 | 103.8693 | 24.23607 |
| WS-QB-3 | 104.3067 | 23.9409 |
| WS-WS-1 | 104.0286 | 23.25986 |
| WS-WS-2 | 104.0286 | 23.25986 |
| WS-WS-3 | 104.105 | 23.24423 |
| WS-XC-1 | 104.6814 | 23.43861 |
| WS-XC-2 | 104.6832 | 23.43965 |
| WS-YS-1 | 104.5804 | 23.74818 |
| WS-YS-2 | 103.9517 | 23.72654 |
| GZ-WN-1 | 103.7602 | 26.77837 |

**Table S2. Environment variables analyzed by the current period model**

| Variable description | Abbreviated name | Unit |
| --- | --- | --- |
| Annual meantemperature | Bio 1 | ℃ |
| Mean diurnal range | Bio 2 | ℃ |
| Isothermality | Bio 3 | - |
| Temperature seasonality | Bio 4 | - |
| Max temperature of warmest month | Bio 5 | ℃ |
| Min temperature of coldest month | Bio 6 | ℃ |
| Temperature annual range | Bio 7 | ℃ |
| Mean temperature of wettest quarter | Bio 8 | ℃ |
| Mean temperature of driest quarter | Bio 9 | ℃ |
| Mean temperature of warmest quarter | Bio 10 | ℃ |
| Mean temperature of coldest quarter | Bio 11 | ℃ |
| Annual precipitation | Bio 12 | mm |
| Precipitation of wettest month | Bio 13 | mm |
| Precipitation of driest month | Bio 14 | mm |
| Precipitation seasonality | Bio 15 | - |
| Precipitation of wettest quarter | Bio16 | mm |
| Precipitation of driest quarter | Bio 17 | mm |
| Precipitation of warmest quarter | Bio 18 | mm |
| Precipitation of coldest quarter | Bio 19 | mm |
| Elevation | Elev | M |
| 12-month average temperatures | TAVG_1~12 | °C |
| 12-month average solar radiations | SRAD_1~12 | kJ m^-2^ day^-1^ |
| 12-month average precipitations | PREC_1~12 | mm |
| Slope | Slo | ° |
| Aspect | Asp | - |
| Topsoil Gravel Content | T_GRAVEL | %vol |
| Topsoil Sand Fraction | T_SAND | % wt |
| Topsoil Silt Fraction | T_SILT | % wt. |
| Topsoil Clay Fraction | T_CLAY | % wt. |
| Topsoil USDA Texture Classification | T_USDA_TEX_CLASS | - |
| Topsoil Reference Bulk Densit | T_REF_BULK_DENSITY | kg/dm^3^ |
| Topsoil Organic Carbon | T_OC | % weight |
| Topsoil pH (H_2_O) | T_PH_H2O | -log(H^+^) |
| Topsoil CEC (clay) | T_CEC_CLAY | cmol/kg |
| Topsoil CEC (soil) | T_CEC_SOIL | cmol/kg |
| Topsoil Base Saturation | T_BS | % |
| Topsoil TEB | T_TEB | cmol/kg |
| Topsoil Calcium Carbonate | T_CACO3 | % weight |
| Topsoil Gypsum | T_CASO4 | % weight |
| Topsoil Sodicity (ESP) | T_ESP | % |
| Topsoil Salinity (Elco) | T_ECE | dS/m |
| Subsoil Gravel Content | S_GRAVEL | %vol |
| Subsoil Sand Fraction | S_SAND | % wt |
| Subsoil Silt Fraction | S_SILT | % wt |
| Subsoil Clay Fraction | S_CLAY | % wt |
| Subsoil USDA Texture Classification | S_USDA_TEX_CLASS | - |
| Subsoil Reference Bulk Density | S_REF_BULK_DENSITY | kg/dm^3^ |
| Subsoil Organic Carbon | S_OC | % weight |
| Subsoil pH (H_2_O) | S_PH_H2O | -log(H^+^) |
| Subsoil CEC (clay) | S_CEC_CLAY | cmol/kg |
| Subsoil CEC (soil) | S_CEC_SOIL | cmol/kg |
| Subsoil Base Saturation | S_BS | % |
| Subsoil TEB | S_TEB | cmol/kg |
| Subsoil Calcium Carbonate | S_CACO3 | % weight |
| Subsoil Gypsum | S_CASO4 | % weight |
| Subsoil Sodicity (ESP) | S_ESP | % |
| Subsoil Salinity (ECE) | S_ECE | dS/m |
| Soil Unit Symbol (FAO-90) | SU_SYM90 | - |
| Topsoil Texture | T_TEXTURE | - |
| Reference Soil Depth | REF_DEPTH | - |
| Drainage class | DRAINAGE | - |

**Table S3. Pearson coefficient between environment variables**

|  | ASPECT | BIO_2 | BIO_3 | BIO_4 | BIO_6 | BIO_7 | PREC_6 | PREC_8 | PREC_10 | REF_DE | S_USDA | SLOPE | SRAD_1 | SRAD_5 | SRAD_6 | SRAD_7 | SRAD_8 | SRAD_9 | SRAD_10 | SU_SYM_90 | T_USDA | TAVG_4 | TAVG_9 |
| --- | --- | --- | --- | --- | --- | --- | --- | --- | --- | --- | --- | --- | --- | --- | --- | --- | --- | --- | --- | --- | --- | --- | --- |
| ASPECT | 1 | 0.089 | 0.025 | 0.025 | -0.131 | 0.068 | 0.036 | -0.144 | -0.067 | -0.027 | 0.043 | -0.042 | -0.012 | -0.055 | -0.08 | 0.071 | 0.095 | 0.08 | 0.024 | -0.042 | 0.043 | -0.109 | -0.096 |
| BIO_2 | 0.089 | 1 | 0.898 | -0.768 | -0.335 | -0.38 | -0.232 | -0.165 | -0.091 | 0.155 | 0.167 | 0.18 | 0.838 | 0.742 | -0.141 | -0.751 | -0.629 | 0.189 | 0.513 | -0.074 | 0.167 | -0.182 | -0.598 |
| BIO_3 | 0.025 | 0.898 | 1 | -0.966 | -0.019 | -0.743 | -0.02 | 0.152 | 0.034 | 0.159 | 0.203 | 0.082 | 0.926 | 0.911 | 0.047 | -0.687 | -0.556 | 0.41 | 0.724 | 0.045 | 0.203 | 0.067 | -0.463 |
| BIO_4 | 0.025 | -0.768 | -0.966 | 1 | -0.16 | 0.875 | -0.097 | -0.336 | -0.121 | -0.133 | -0.184 | -0.037 | -0.905 | -0.94 | -0.13 | 0.629 | 0.511 | -0.471 | -0.773 | -0.094 | -0.184 | -0.206 | 0.344 |
| BIO_6 | -0.131 | -0.335 | -0.019 | -0.16 | 1 | -0.444 | 0.375 | 0.557 | 0.324 | -0.068 | 0.001 | -0.308 | 0.087 | 0.233 | 0.43 | 0.272 | 0.308 | 0.38 | 0.306 | 0.229 | 0.001 | 0.964 | 0.839 |
| BIO_7 | 0.068 | -0.38 | -0.743 | 0.875 | -0.444 | 1 | -0.338 | -0.582 | -0.185 | -0.084 | -0.151 | 0.085 | -0.668 | -0.795 | -0.288 | 0.325 | 0.24 | -0.565 | -0.744 | -0.184 | -0.151 | -0.396 | 0.094 |
| PREC_06 | 0.036 | -0.232 | -0.02 | -0.097 | 0.375 | -0.338 | 1 | 0.413 | 0.573 | -0.049 | 0.156 | -0.067 | 0.02 | 0.098 | 0.366 | 0.472 | 0.536 | 0.687 | 0.482 | 0.305 | 0.156 | 0.276 | 0.302 |
| PREC_08 | -0.144 | -0.165 | 0.152 | -0.336 | 0.557 | -0.582 | 0.413 | 1 | 0.379 | 0.041 | -0.089 | -0.084 | 0.127 | 0.356 | 0.387 | -0.039 | -0.12 | 0.229 | 0.357 | 0.171 | -0.089 | 0.46 | 0.29 |
| PREC_10 | -0.067 | -0.091 | 0.034 | -0.121 | 0.324 | -0.185 | 0.573 | 0.379 | 1 | -0.017 | 0.236 | 0.034 | 0.227 | 0.22 | 0.263 | 0.203 | 0.286 | 0.543 | 0.437 | 0.246 | 0.236 | 0.246 | 0.296 |
| REF_DE | -0.027 | 0.155 | 0.159 | -0.133 | -0.068 | -0.084 | -0.049 | 0.041 | -0.017 | 1 | -0.149 | 0.077 | 0.13 | 0.124 | 0.028 | -0.122 | -0.121 | 0.01 | 0.083 | -0.013 | -0.149 | -0.049 | -0.132 |
| S_USDA | 0.043 | 0.167 | 0.203 | -0.184 | 0.001 | -0.151 | 0.156 | -0.089 | 0.236 | -0.149 | 1 | 0.039 | 0.232 | 0.13 | -0.094 | -0.049 | 0.036 | 0.256 | 0.215 | 0.375 | 1 | -0.004 | -0.08 |
| SLOPE | -0.042 | 0.18 | 0.082 | -0.037 | -0.308 | 0.085 | -0.067 | -0.084 | 0.034 | 0.077 | 0.039 | 1 | 0.084 | 0.039 | -0.252 | -0.294 | -0.302 | -0.138 | -0.039 | -0.081 | 0.039 | -0.304 | -0.275 |
| SRAD_1 | -0.012 | 0.838 | 0.926 | -0.905 | 0.087 | -0.668 | 0.02 | 0.127 | 0.227 | 0.13 | 0.232 | 0.084 | 1 | 0.937 | 0.126 | -0.635 | -0.481 | 0.51 | 0.809 | 0.107 | 0.232 | 0.165 | -0.28 |
| SRAD_5 | -0.055 | 0.742 | 0.911 | -0.94 | 0.233 | -0.795 | 0.098 | 0.356 | 0.22 | 0.124 | 0.13 | 0.039 | 0.937 | 1 | 0.32 | -0.567 | -0.459 | 0.516 | 0.837 | 0.129 | 0.13 | 0.287 | -0.196 |
| SRAD_6 | -0.08 | -0.141 | 0.047 | -0.13 | 0.43 | -0.288 | 0.366 | 0.387 | 0.263 | 0.028 | -0.094 | -0.252 | 0.126 | 0.32 | 1 | 0.468 | 0.428 | 0.554 | 0.522 | 0.282 | -0.094 | 0.356 | 0.341 |
| SRAD_7 | 0.071 | -0.751 | -0.687 | 0.629 | 0.272 | 0.325 | 0.472 | -0.039 | 0.203 | -0.122 | -0.049 | -0.294 | -0.635 | -0.567 | 0.468 | 1 | 0.964 | 0.284 | -0.149 | 0.225 | -0.049 | 0.165 | 0.526 |
| SRAD_8 | 0.095 | -0.629 | -0.556 | 0.511 | 0.308 | 0.24 | 0.536 | -0.12 | 0.286 | -0.121 | 0.036 | -0.302 | -0.481 | -0.459 | 0.428 | 0.964 | 1 | 0.432 | -0.023 | 0.263 | 0.036 | 0.223 | 0.519 |
| SRAD_9 | 0.08 | 0.189 | 0.41 | -0.471 | 0.38 | -0.565 | 0.687 | 0.229 | 0.543 | 0.01 | 0.256 | -0.138 | 0.51 | 0.516 | 0.554 | 0.284 | 0.432 | 1 | 0.875 | 0.401 | 0.256 | 0.331 | 0.163 |
| SRAD_10 | 0.024 | 0.513 | 0.724 | -0.773 | 0.306 | -0.744 | 0.482 | 0.357 | 0.437 | 0.083 | 0.215 | -0.039 | 0.809 | 0.837 | 0.522 | -0.149 | -0.023 | 0.875 | 1 | 0.302 | 0.215 | 0.293 | -0.034 |
| SU_SYM_90 | -0.042 | -0.074 | 0.045 | -0.094 | 0.229 | -0.184 | 0.305 | 0.171 | 0.246 | -0.013 | 0.375 | -0.081 | 0.107 | 0.129 | 0.282 | 0.225 | 0.263 | 0.401 | 0.302 | 1 | 0.375 | 0.188 | 0.173 |
| T_USDA | 0.043 | 0.167 | 0.203 | -0.184 | 0.001 | -0.151 | 0.156 | -0.089 | 0.236 | -0.149 | 1 | 0.039 | 0.232 | 0.13 | -0.094 | -0.049 | 0.036 | 0.256 | 0.215 | 0.375 | 1 | -0.004 | -0.08 |
| TAVG_4 | -0.109 | -0.182 | 0.067 | -0.206 | 0.964 | -0.396 | 0.276 | 0.46 | 0.246 | -0.049 | -0.004 | -0.304 | 0.165 | 0.287 | 0.356 | 0.165 | 0.223 | 0.331 | 0.293 | 0.188 | -0.004 | 1 | 0.821 |
| TAVG_9 | -0.096 | -0.598 | -0.463 | 0.344 | 0.839 | 0.094 | 0.302 | 0.29 | 0.296 | -0.132 | -0.08 | -0.275 | -0.28 | -0.196 | 0.341 | 0.526 | 0.519 | 0.163 | -0.034 | 0.173 | -0.08 | 0.821 | 1 |

**Table S4. Data regarding the modeling environment variables**

| Variable | Percent contribution | Permutation importance |
| --- | --- | --- |
| bio_7 | 32.4 | 46.4 |
| srad_6 | 22.7 | 8.6 |
| srad_7 | 18.3 | 1.4 |
| srad_10 | 8.6 | 20.4 |
| prec_8 | 8.1 | 12.8 |
| bio_4 | 2.6 | 0.1 |
| S_USDA | 2.1 | 2.2 |
| prec_6 | 1.6 | 0.7 |
| tavg_4 | 1.3 | 0.5 |
| bio_2 | 1.2 | 5.8 |
| aspect | 0.6 | 0.7 |
| SU_SYM90 | 0.4 | 0.2 |
| slope | 0.2 | 0 |
